# Diversity Under Pressure: Long‐Term Genetic Decline in California's Central Valley Chinook Salmon ESA Listed Runs

**DOI:** 10.1002/ece3.74176

**Published:** 2026-08-13

**Authors:** E. E. Collins, T. Q. Thompson, P. Goertler, M. R. Baerwald, M. H. Meek

**Affiliations:** ^1^ Department of Integrative Biology Michigan State University East Lansing Michigan USA; ^2^ Michigan State University, Evolution, Ecology, and Behavior Program, Biomedical & Physical Sciences Building East Lansing Michigan USA; ^3^ Wild Salmon Center Portland Oregon USA; ^4^ California Department of Water Resources, Division of Integrated Science and Engineering Sacramento California USA; ^5^ California Department of Water Resources, Division of Environmental Services West Sacramento California USA; ^6^ The Wilderness Society Bozeman Montana USA

**Keywords:** conservation, effective population size, genetics, historical change, salmon

## Abstract

Intraspecific diversity enables populations to persist under stochastic and extreme environmental conditions. One system that is representative of notable intraspecific diversity is Chinook salmon ( *Oncorhynchus tshawytscha* ) in the Central Valley of California, USA. It is the only place in the species range where four distinct migration timings (Winter, Spring, Fall, Late‐fall) co‐occur. These populations are declining, with Winter run listed as Endangered and Spring run as Threatened under the Endangered Species Act (ESA). To quantify temporal changes in genetic diversity of the different runs, we genotyped outmigrating juveniles using RAD‐sequencing across 20+ years of annual sampling. Tajima's D revealed significant shifts in neutral genetic variation over time in Spring and Winter runs. Effective population size (*Ne*) declined in all listed populations, and the historical *Ne* estimates indicate severe plummets in genetic diversity 25–50 generations ago. Our results demonstrate how anthropogenic forces have eroded the genetic diversity of ESA listed Chinook salmon populations over the past century. Moreover, this study demonstrates how an organism with genetically based life history variation (migration timing), traditionally advantageous under natural environmental variability, struggles to persist when faced with anthropogenically altered habitat and changing climate.

## Graphical abstract

Chinook salmon in the Central Valley of California have notable intraspecific diversity because it is the only place where four distinct migration timings (Winter, Spring, Fall, Late‐fall) co‐occur. The Winter run is listed as Endangered and the Spring run is listed as Threatened under the Endangered Species Act. To quantify temporal changes in genetic diversity of the different migrations, we genotyped individuals across 20+ years of annual sampling. Effective population size (*Ne*) declined in all listed populations, and the historical *Ne* estimates indicate severe plummets in genetic diversity 25–50 generations ago.

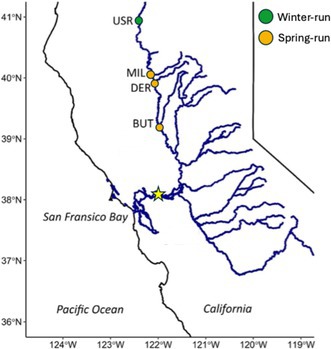

## 1 Introduction

Humans have fished for tens of thousands of years (Hu et al. 2009), but relatively recent increases in fishing intensity have led to most fish stocks today being fully exploited or overexploited (FAO 2020; Froese et al. 2012; Jackson et al. 2001; Jennings and Blanchard 2004; Pauly et al. 2002; Pitcher 2001). Accurate stock assessments require historical abundance data, but such data are often lacking. For some species of interest in the United States, historical fisheries records may begin around the middle of 19th century. This information gap contributes to the “shifting baseline syndrome”, a gradual, often unnoticed decline in population baselines over time (Pauly 1995; McClenachan et al. 2012; Guerrero‐Gatica et al. 2019; Lotze and Worm 2009; Préfontaine 2009). The consequences of this shifting baseline can obscure true stock status and lead to misinformed management and conservation decisions (Jackson et al. 2011; McClenachan et al. 2012; Kittinger et al. 2015; Brown and Trebilco 2014). As a result, fish populations that haven't been the subject of long‐term monitoring efforts may undergo unnoticed changes in abundance, age structure, geographic range, and genetic diversity (Berkeley et al. 2004; Rouyer et al. 2011).

Given that long‐term census data are unavailable for many populations, genetic approaches provide an alternative means of inferring demographic change. One such metric is effective population size (*Ne*), which measures the loss of genetic diversity driven by evolutionary processes such as genetic drift and natural selection (Waples 2022; Mamoozadeh et al. 2025). Although *Ne* is often correlated with census abundance (*Nc*), it reflects the number of individuals effectively contributing genes to subsequent generations and is therefore more closely related to reproductive dynamics than to total population size. Consequently, *Ne* is typically lower than census size, with the relationship between *Ne* and *Nc* varying among species and populations (Kalinowski and Waples 2002). Effective population size can be estimated using several approaches, including temporal or contemporary, assessed through linkage disequilibrium (LD) or coalescent‐based methods, each operating over different time scales and with distinct assumptions (Nadachowska‐Brzyska et al. 2022; Mamoozadeh et al. 2025). *Ne* also offers a genomic approach to detecting population changes that census data alone may miss, such as variance in reproductive success, fluctuations in population sizes over time, or non‐random mating. A limitation of this metric is population size; estimation can be difficult for very small populations, where sampling variance is high, and for very large populations, where genetic drift signals become weak. It is important to note that *Ne* is a demographic parameter rather than a direct measure of genetic diversity; therefore, it is most informative when considered alongside genetic diversity metrics.

Recognized as a valuable biodiversity indicator, the *Ne* statistic has been increasingly estimated by managers to inform species management decisions and for major policy decisions, such as the Kunming–Montreal Global Biodiversity Framework (Hoban et al. 2022; Hoban et al. 2024). *Ne* has been proposed as a standardized biodiversity monitoring metric and has been commonly implemented through the rule of 100/1000 to identify populations at high risk of extirpation if *Ne* estimates fall below 100 and low risk of extirpation if *Ne* rates exceed 1000 (Frankham et al. 2014; Hoban et al. 2023; Robuchon et al. 2023; Schmidt et al. 2023; Hoban et al. 2024). *Ne* estimates can provide insights about changes in populations across generations, which are particularly useful under rapid environmental change (Hoban et al. 2022). For example, *Ne* has guided management in multiple species, such as Arctic charr, where declining *Ne* in southern populations highlighted climate‐driven impacts at the edge of the species' range (Layton et al. 2021). Similarly, for commercially harvested Australian gummy sharks, *Ne* estimates revealed a recent decline in the genetic health of one population isolated by the East Australian Current and increasingly vulnerable to climate‐related shifts in food availability and habitat (Petrolo et al. 2021). In Chinook salmon ( *Oncorhynchus tshawytscha* ), Shrimpton and Heath (2003) demonstrated population declines with *Ne* estimates that would have been undetected by census estimates alone.

While *Ne* provides insight into the demographic processes shaping populations through time, its conservation relevance stems largely from its relationship with genetic diversity as a critical indicator of population resilience. Genetic diversity enables populations to adapt to variable and extreme environmental conditions (May 1994; Pauls et al. 2013; Messer et al. 2016), yet it is declining globally (Leigh et al. 2019; Hoban et al. 2021; Exposito‐Alonso et al. 2022). Reductions in population and genetic diversity can erode the portfolio of diversity required for resilience to environmental fluctuations (Hilborn et al. 2003; Schindler et al. 2010; Schijns and Pauly 2022). Grounding conservation targets in pre‐industrial population sizes and genetic diversity can help avoid the accidental loss of distinct local populations that enhance species‐wide fitness and combat shifting baseline syndrome (Hilborn et al. 2003; Schindler et al. 2010; Mimura et al. 2017). This is especially important for keystone species whose declines can disrupt entire ecosystems.

Chinook salmon are a valuable model for studying genetic diversity loss over time due to their role as a keystone species, diverse life history strategies, and substantial population changes since pre‐colonial times (Atlas et al. 2023). Migration timing, a heritable trait with direct fitness consequences, is a key component of Chinook salmon life history (Waples et al. 2022). Chinook salmon migration timing varies seasonally and by habitat, with several distinct runs potentially occurring within a single river system. Adaptation to local environmental conditions over millennia has led to the evolution of these specialized lineages (Allendorf et al. 1987; Quinn 2021).

**FIGURE 1:**
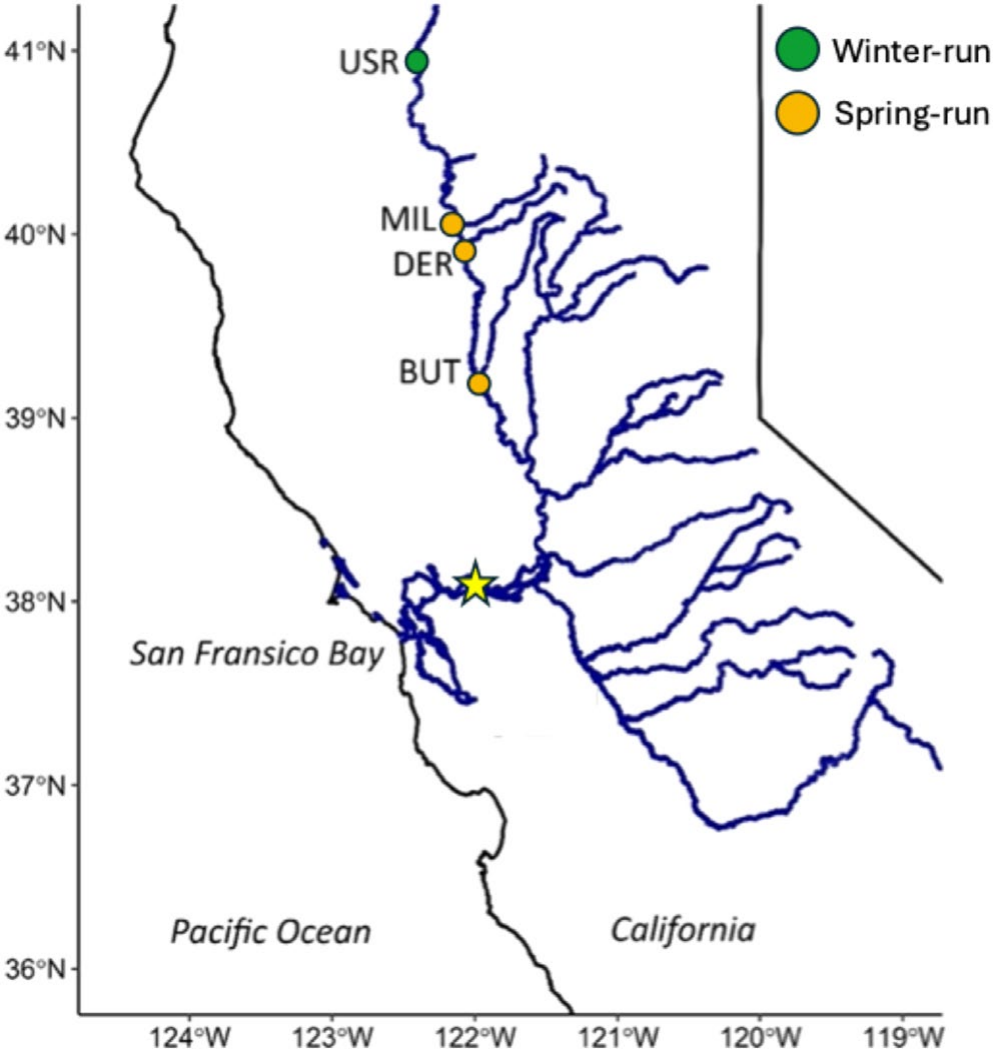
A map of the sample region modified from O'Leary et al. (2024). Chipps Island is marked with a star and the other locations include: USR (Upper Sacramento River), MIL (Mill Creek), DER (Deer Creek), and BUT (Butte Creek). The marked locations are where samples were collected for this study and where samples were collected for the genetic assignment baseline. The hatchery‐origin samples are squares and the natural‐origin are circles. The yellow symbols are Spring run and the green symbols are Winter run.

Across much of the Chinook salmon range, two primary migration timings, Spring and Fall, are observed. California's Central Valley (CV), however, exhibits exceptional diversity, with four distinct runs, all named for the time they return to freshwater to spawn: Winter, Spring, Fall, and Late‐Fall (Figure 1; Williams 2006). These runs are genetically distinct (Meek et al. 2016, 2020; Nelson et al. 2022; Thompson et al. 2024), with the migration timing difference between early (Winter and Spring) and late (Fall and Late‐Fall) runs associated with a major effect locus near the *GREB1L* gene (Narum et al. 2018; Prince et al. 2017; Thompson et al. 2020). California's CV also experiences the most extreme interannual climatic variability within the Chinook salmon range, potentially driving the evolution of these migration timings (Dettinger 2011; Swain et al. 2018). These recent genetic discoveries related to migration timing align with Indigenous knowledge of “seasonal rounds” of Chinook salmon (e.g., Carney et al. 2021; Matsaw 2020).

Salmon conservation is a cultural value for Indigenous peoples of the region, who have long observed and passed down knowledge about Chinook salmon lineages, including their distribution, abundance, and seasonal timing (Quaempts et al. 2018; FiveCrows et al. 2023). In the 1800s, settlers along the West Coast began harvesting Chinook salmon without the traditional knowledge and sustainable practices of the Indigenous peoples, leading to population declines (FiveCrows et al. 2023). Today's Chinook salmon CV populations are threatened by post‐industrial human impacts such as impassable dams, hatchery practices, and climate change. Dams have blocked access to all historical Winter run habitat and early dams and mining led to the extirpation of Spring run in most rivers (Williams 2006). The declines caused by these anthropogenic impacts have led to Winter run being listed as Endangered (NOAA, FR 1994) and the Spring run as Threatened under the Endangered Species Act (NOAA, FR 2005). These declines are especially concerning given the cultural, ecological, and economic importance of Chinook salmon (Fisher et al. 1991), underscoring the urgency of conserving their genetic diversity for long‐term population persistence. These recent declines highlight the importance of reconstructing past population abundances so we can better evaluate what a healthy and fully recovered population should look like today.

In this study, we used genomic data from samples collected from Chinook salmon in the Central Valley over a 20‐year period to ask: (1) Have there been changes in genetic variation in the ESA‐listed Spring and Winter run populations over time? and (2) What is the magnitude of changes in *Ne* over time and how do observed changes correlate with the timeline of major anthropogenic influences in the region? Beyond direct conservation implications for these imperiled populations and a better understanding of the historical abundance these populations once had, this study highlights the critical importance of identifying and monitoring changes in intraspecific genetic diversity in organisms to detect declining trends before irreversible genetic diversity loss occurs.

## 2 Materials and Methods

To evaluate genetic diversity and effective population sizes in CV Spring and Winter run Chinook salmon over the last 20 years, we utilized data from a previously published study (Thompson et al. 2024). The objective of the Thompson et al. (2024) study was to build a reliable baseline to assign Chinook salmon samples collected from the CV to run timing populations and this differs from the objective of this study to track shifts in neutral genetic variation and effective population size over time.

### 2.1 Sample Origin and Initial Data Processing

All Chinook salmon in the CV are spawned in tributaries to the Sacramento and San Joaquin Rivers and migrate out to the ocean as juveniles through the CV Delta, passing by Chipps Island. We utilized archived samples collected from Chipps Island during the juvenile outmigration over a twenty‐year period (1996–2018) to evaluate intraspecific‐level diversity in listed CV Chinook salmon (Figure 1). The archived samples were collected via midwater trawling; conducted in ten iterations of 20‐min, 3–7 days per week, within a 3 km reach of Chipps Island (Brandes and McLain 2001; Pyper et al. 2013).

The subpopulations of interest are the Mill Creek, Deer Creek, and Butte Creek populations within the Spring run (Figure 1). The outmigrating juvenile Chinook salmon collected for this study were sampled from the territories of Miwok, Patwin, Me‐Wuk (Bay Miwok), and the Confederated Villages of Lisjan and the individuals collected were spawned across the Central Valley, which covers the territory of over 100 tribes (Native Land Digital).

This dataset genetically sequenced approximately 622 Chinook salmon juveniles sampled while outmigrating passed Chipps Island in the lower Sacramento River Delta (Figure 1) using a RAD‐sequencing protocol (Ali et al. 2016), then assigned each sample to one of the major demographic groups in the CV (Winter, Spring, Fall, and Late‐Fall runs), as well as to subpopulations within the Spring run (Mill/Deer Creek, and Butte Creek; Figure 1). In total, 325 samples confidently assigned to the Spring run (159 Mill/Deer and 166 Butte Creek) and 220 samples confidently assigned to the Winter run, and these samples were included in the current study.

### 2.2 Molecular Methods and Data Filtering

**FIGURE 2:**
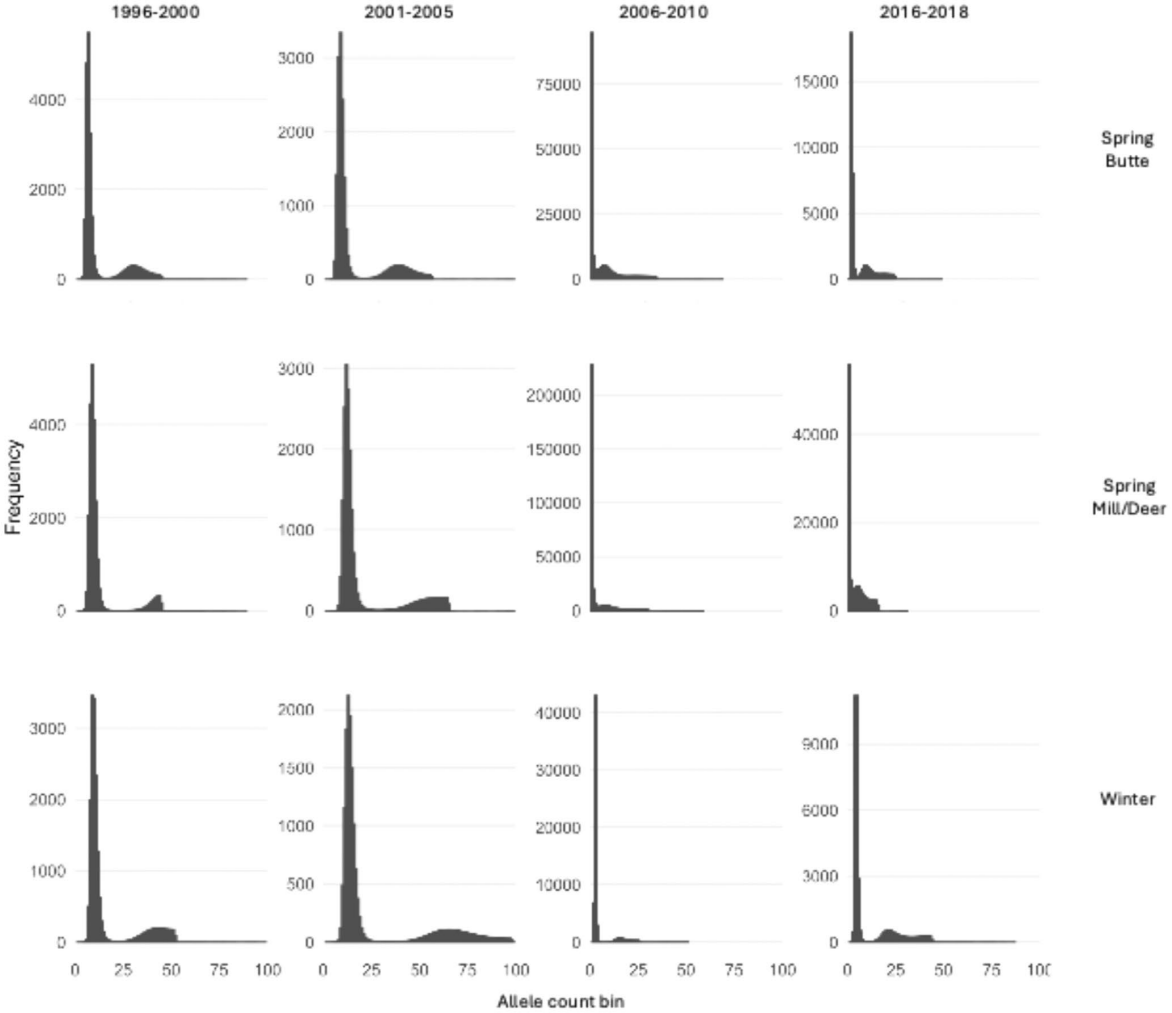
Site frequency spectrum (SFS). The vertical axis on the left measures the frequency values for each value on the spectrum and the vertical axis on the right notes the run timing population analyzed. The horizontal axis is divided into plots by the collection years and represents the allele count bins.

Sequencing data alignment and initial processing was conducted with the same methods and parameters as described in Thompson et al. (2024). Briefly, raw fastq files were mapped to the Chinook salmon reference genome (Otsh_v2.0; GCA_018296145.1; Christensen et al. 2018) with bwa‐mem (Vasimuddin et al. 2019) and samtools (Danecek et al. 2021) was used to quality filter the mapped reads. Only uniquely mapped, properly paired reads with mapping and base quality scores > 30 were retained. Samples with fewer than 1000 final aligned reads after filtering were excluded from downstream analyses. Thompson et al. (2024) identified a panel of single nucleotide polymorphisms (SNPs) present in at least 50% of samples from that study with minor allele frequencies > 0.05, and that panel of SNPs was used for analyses in this current study. We filtered the samples by missingness by population according to the Thompson et al. (2024) Table S2. To get the most information from the 20 years of samples with varying quality, Thompson et al. (2024) sampled a single allele at each SNP locus instead of calling a genotype with ANGSD (‐doIBS 1, ‐doCounts 1) (Korneliussen et al. 2014). This dataset of 4632 SNP sites is what was used in this study for all subsequent analyses. Genotype likelihoods were computed using the SAMtools model (−GL 1); site allele frequency likelihoods were generated with ‐doSaf 1 to produce site allele frequency likelihood (.saf) files, and the global SFS was inferred using the realSFS tool, which computes the maximum likelihood estimate from these files (Figure 2). Only sites with strong evidence of polymorphism were retained (SNP *p*‐value ≤ 1 × 10^−6^; ‐SNP_pval 1e‐6). We accounted for inflated singletons caused by low coverage and sequencing errors by applying stringent filters. We increased quality thresholds (−minMapQ 30, −minQ 30, −baq 1) to reduce false SNP calls, enabled error modeling (‐doCounts 1, ‐doError 1), and filtered by depth (‐setMinDepth 8, ‐setMaxDepth 60) to exclude sites with extreme coverage.

### 2.3 Analyses

GONE (Santiago et al. 2020) estimates past *Ne* by algorithmically selecting for the demographic scenario that provides the best fit to the observed LD patterns among sampled individuals. We used GONE with default settings, including 40 replicates, to estimate current and historic *Ne* values. The generations began at 100, which represented *Ne* values 100 generations into the past and moved forward in time until reaching 0 generations, which represented the current *Ne* values. Unlike estimators that focus only on contemporary *Ne*, GONE analyzes LD patterns across a wide range of genetic distances and recombination rates to reconstruct *Ne* trajectories over past generations, enabling detection of complex demographic histories with repeated bottlenecks, declines, and expansions (Mitchell 1998; Hayes et al. 2003; Nadachowska‐Brzyska et al. 2022). This approach is highly versatile; simulation analyses conducted during the development of GONE indicated that the method can recover recent demographic changes from samples containing fewer than ten individuals derived from low‐coverage sequencing data, although uncertainty is expected to increase as sample size and genotype quality decrease (Santiago et al. 2020). To place historic *Ne* values in context, we combed the literature to add major anthropogenic impacts to Chinook salmon populations in the CV to the timeline.

Genetic diversity statistics were calculated using ANGSD (Korneliussen et al. 2014). As detailed in the methods, the site frequency spectrum (SFS) was estimated from genotype likelihoods rather than called genotypes to accommodate lower depth sequencing data. Tajima's D was calculated using SFS‐based estimates of genetic diversity by comparing nucleotide diversity (π) and Watterson's θ. Per‐site θ estimates were obtained with saf2theta within the realSFS tool using the .saf.idx file and global SFS, and summary statistics including π, θ π, Watterson's θ, Fay & Wu's H, and Tajima's D were computed across windows using thetaStat do_stat. To assess whether changes in Tajima's D distributions over time were significant, we applied Kolmogorov–Smirnov (K–S) tests to compare cumulative distributions and Chi‐squared (*χ* ^2^) tests to evaluate differences in binned frequency counts (e.g., D < −2, −2 to 0, 0 to 2). These tests provide information on whether observed shifts in allele frequency patterns deviate from neutral expectations and reflect demographic changes or selection rather than random variation, although such deviations cannot be uniquely attributed to selection or demographic processes.

To further validate our findings from the GONE analyses, we also employed NeEstimator for a contemporary *Ne* estimate. We used NeEstimator v2.1 (Do et al. 2014) with default settings to reveal changes to *Ne* over the 20‐year collection period. We applied the single‐sample LD method, which infers *Ne* from nonrandom associations among alleles across the genome. To minimize bias from small sample sizes, a known sensitivity of LD‐based estimates, Spring run subpopulations (Butte Creek and Mill/Deer Creeks) were pooled following best practices for LD‐based estimation (Beebee 2009). We also combined consecutive years into three periods (1996–2007, 2008–2015, and 2016–2018) to increase sample sizes, improve reliability, and narrow confidence intervals. This pooling strategy assumes that allele frequencies and demographic processes remained relatively stable within each period, as combining multiple cohorts can bias *Ne* estimates when substantial temporal genetic change occurs. These periods also reflect major environmental differences: variable precipitation during 1996–2007, two severe statewide droughts contained within 2008–2015, and extensive flooding in the Yolo Bypass, a key migration corridor for Central Valley Chinook salmon, between 2016 and 2018. Environmental conditions influence all life stages, and non‐natal rearing habitats are particularly critical for the Winter run population (Phillis et al. 2018). Although other single‐sample estimators exist, such as those based on sibship frequency (Wang 2009, 2016, 2025; Waples 2021), heterozygote excess (Luikart and Cornuet 1999; Pudovkin et al. 1996, 2010), and molecular coancestry (Nomura 2008), LD‐based methods are frequently applied because they are computationally efficient, require only a single sampling event, are readily implemented with genomic datasets, and have been shown to provide robust estimates of contemporary *Ne* under a wide range of conditions (Hill 1981; Waples 2006; Waples and Do 2010).

## 3 Results

### 3.1 Estimation of Historical Changes in *Ne*

**FIGURE 3:**
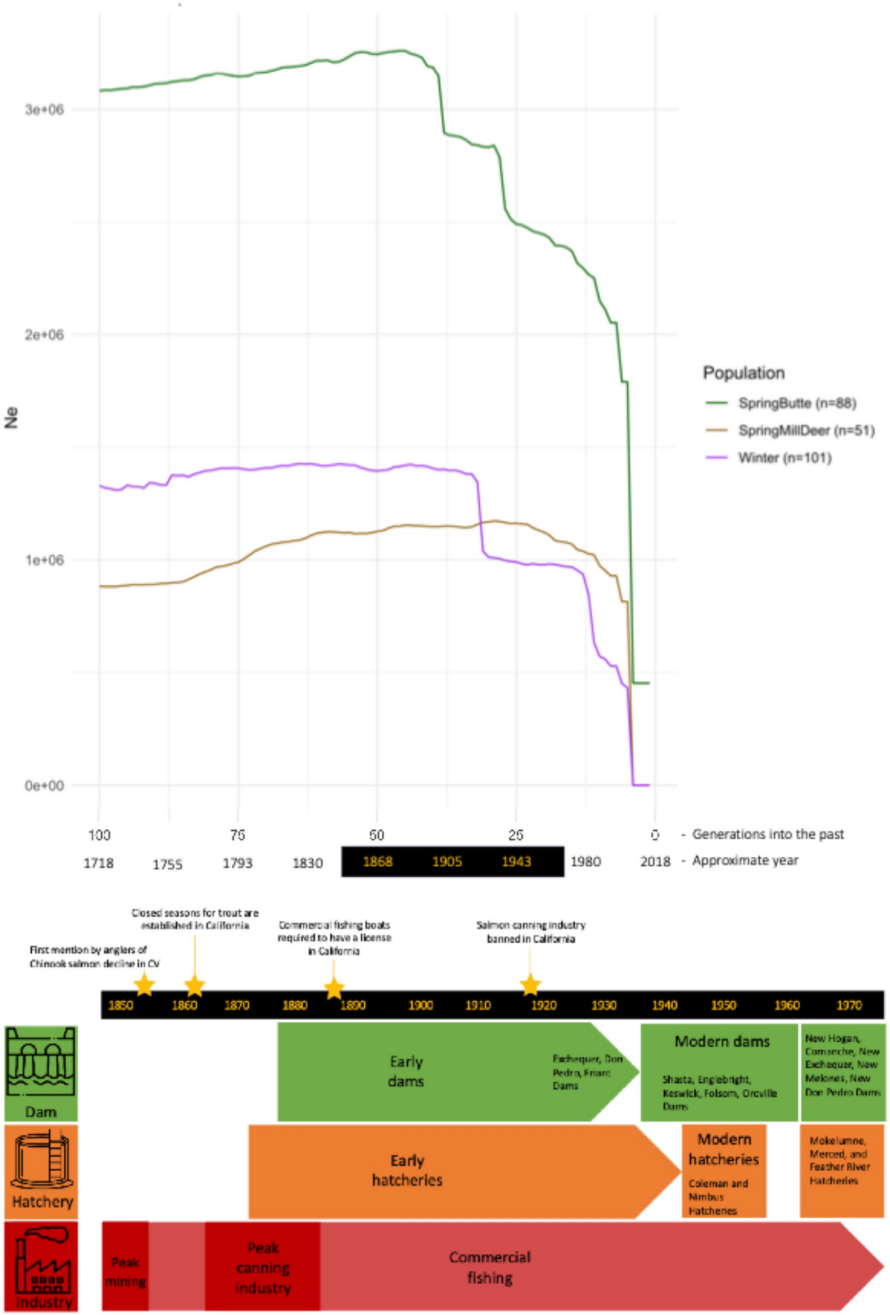
Line plots of effective population size (*Ne*) estimates backward in time showed crashes in *Ne* estimates beginning before samples were collected for this study. *Ne* values are reported on the *y*‐axis and the number of generations into the past is reported on the *x*‐axis. The generations begin at 100, which represents *Ne* values 100 generations into the past and moves forward in time until reaching 0 generations, which represents the current *Ne* values. Each line represents a run timing population and the number of samples is reported in the legend. A timeline of historical impacts to Chinook salmon in the Central Valley is included. The number of generations into the past from the historical *Ne* line plot is matched to years using a Chinook salmon generation of 3 years as an example. Chinook salmon lifespans can vary and overlap within a population; thus the generations translated to years contain variation from the example provided.

Historical *Ne* values were estimated for each population. Our results revealed that Spring and Winter run Chinook salmon populations in the Central Valley were considerably higher in the past (Figure 3). These historic baselines, estimated from 1720 to 1870 (50–100 generations ago), appear to reflect periods of greater genetic health. Among them, the Butte Creek Spring run population stands out with *Ne* values exceeding millions (Figure 3). Around 1870 (50 generations ago), a decline in the Spring run Butte Creek population and the Winter run population began. This decline was followed by the peak of the mining and canning industries in the CV. Preceding the 1900s (30 generations ago), the Winter run population had a greater *Ne* estimate than the Spring run Mill/Deer Creeks population. During the early 1900s (25–30 generations ago), the Winter run population subsequently fell to the lowest *Ne* estimate when modern dams and hatcheries took hold. Around 1940 (25 generations ago), *Ne* estimates for the Spring run Mill/Deer Creeks population began to decline and the decline of all populations became more precipitous (Figure 3). The Spring run Butte Creek population began with the highest *Ne* estimates and had the most drastic crash of all populations.

### 3.2 Neutrality Tests

**FIGURE 4:**
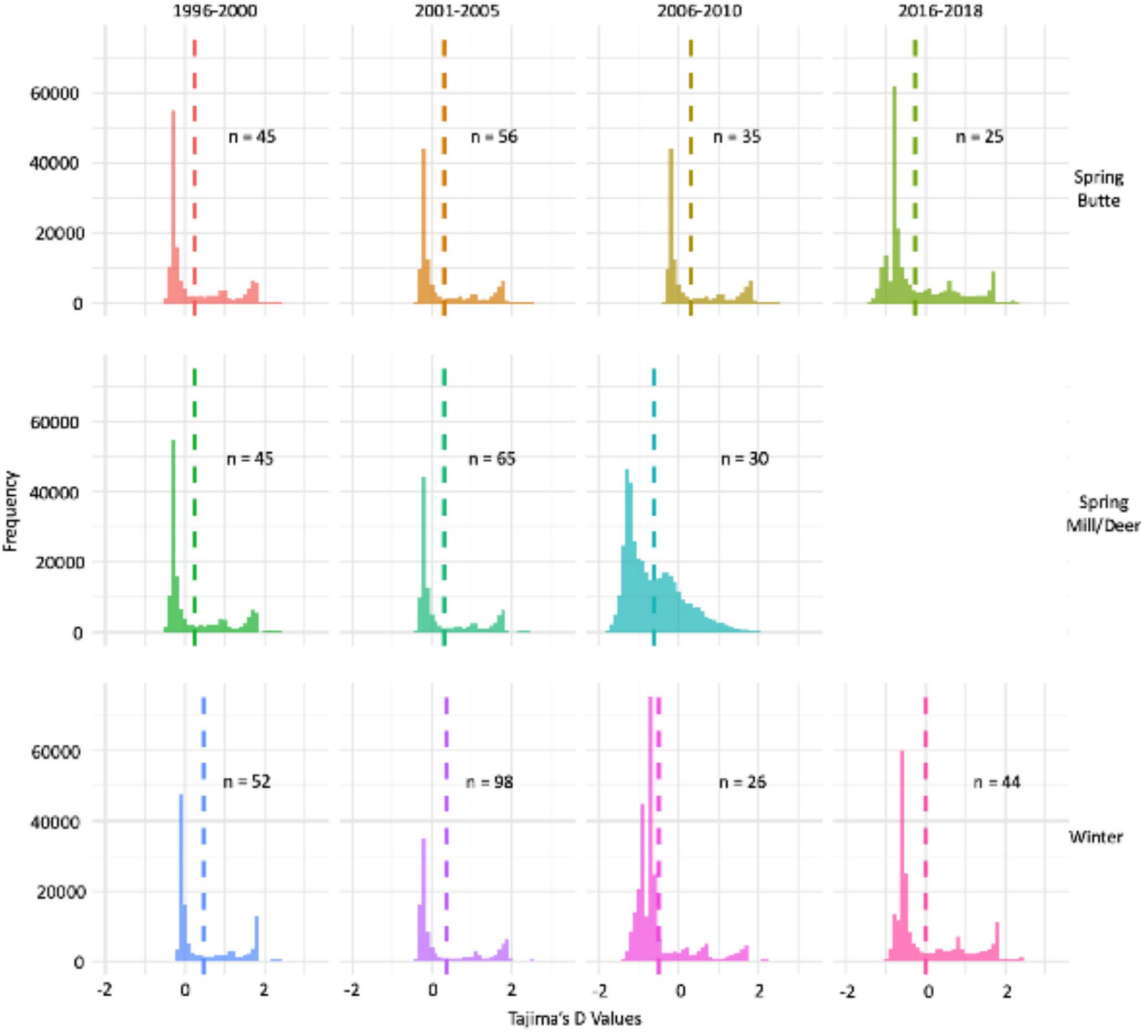
Histograms of Tajima's D values with all Tajima's D values of zero filtered out showed evidence of selection pressures and loss of genetic diversity over time. The *y*‐axis of the histograms represents the number of SNPs that have a particular Tajima's D value or the frequency of each Tajima's D value and the *x*‐axis displays the Tajima's D values. The histograms are arranged in rows for each run timing population and in columns by sample collection years. The number of samples in each Tajima's D analysis is listed within each histogram. The mean Tajima's D values for each histogram are indicated by the dotted lines. Kolmogorov–Smirnov (KS) and Chi‐squared tests were conducted to test for significant differences between the distributions and they are all significantly different with the exception of the 2001–2005 and 2006–2010 Spring Butte distributions.

All populations showed right‐skewed Tajima's D value distributions due to an excess of negative Tajima's D values, which is a signal of lower genetic variation than expected under neutral processes (Table 1; Figure 4; Tajima 1989). Consistent with this pattern, the average Tajima's D value decreased over time in all populations (Table 1). Within the Spring run, samples collected from Butte Creek from 2001–2005 and 2006–2010 were significantly different from one another (Table 1; Figure 4). Correspondingly, Spring run populations exhibited a sharp decline in average π, θπ, Watterson's θ, and Fay & Wu's H between 2006 and 2010, followed by a return to earlier levels between 2016 and 2018 (Table 2). The Winter run population showed a similar temporal pattern, with declines in average π, θπ, and Fay & Wu's H across the sampled years that were most pronounced between 2006 and 2010; however, Watterson's θ remained relatively stable over time (Table 2). The site frequency spectrum (SFS) also reflected the pattern of an excess of low frequency alleles across populations that was more pronounced between 2006 and 2010, consistent with negative Tajima's D values and other diversity statistics (Figure 2).

**TABLE 1:** Tajima's D distributions, averages, significant differences, and number of samples are listed according to run timing population and sample collection years.

Population and year range	Distribution	Average	Sample number (*n*)
Spring Butte 1996–2000	Right skewed	0.23	45
Spring Butte 2001–2005	Right skewed	0.30	56
Spring Butte 2006–2010	Right skewed	0.30	35
Spring Butte 2011–2015	None	NA	5
Spring Butte 2016–2018	Right skewed	−0.26	25
Spring Mill/Deer 1996–2000	Right skewed	0.23	45
Spring Mill/Deer 2001–2005	Right skewed	0.30	65
Spring Mill/Deer 2006–2010	Right skewed	−0.63	30
Spring Mill/Deer 2011–2015	None	NA	3
Spring Mill/Deer 2016–2018	None	NA	16
Winter 1996–2000	Right skewed	0.46	52
Winter 2001–2005	Right skewed	0.36	98
Winter 2006–2010	Right skewed	−0.51	26
Winter 2011–2015	None	NA	0
Winter 2016–2018	Right skewed	0.02	44

*Note:* These results were visualized in histograms in Figure 3. All run timings revealed right‐skewed Tajima's D value distributions. The average Tajima's D value decreased from 0.31 in 1996–2000 to −0.17 in the 2016–2018 samples. Populations with less than 5 samples were excluded from this analysis, which were the 2011–2015 years for all populations. All Tajima's D results were significantly different from one another, except for the 2001–2005 and 2006–2010 Spring run Butte Creek samples.

**TABLE 2:** Nucleotide diversity (π), θπ, Watterson's θ, Fay & Wu's H averages and number of samples are listed according to run timing population and sample collection years.

Population and year range	π	θ π	Watterson's θ	Fay & Wu's H	Sample number (*n*)
Spring Butte 1996–2000	0.23	0.23	0.20	0.09	45
Spring Butte 2001–2005	0.24	0.24	0.19	0.09	56
Spring Butte 2006–2010	0.00	0.00	0.00	0.00	35
Spring Butte 2011–2015	NA	NA	NA	NA	5
Spring Butte 2016–2018	0.19	0.19	0.22	0.08	25
Spring Mill/Deer 1996–2000	0.23	0.23	0.20	0.08	45
Spring Mill/Deer 2001–2005	0.25	0.25	0.18	0.11	65
Spring Mill/Deer 2006–2010	0.00	0.00	0.00	0.00	30
Spring Mill/Deer 2011–2015	NA	NA	NA	NA	3
Spring Mill/Deer 2016–2018	NA	NA	NA	NA	16
Winter 1996–2000	0.26	0.26	0.19	0.12	52
Winter 2001–2005	0.23	0.23	0.17	0.10	98
Winter 2006–2010	0.16	0.16	0.22	0.05	26
Winter 2011–2015	NA	NA	NA	NA	0
Winter 2016–2018	0.21	0.21	0.20	0.09	44

*Note:* Populations with less than 5 samples were excluded from this analysis, which were the 2011–2015 years for all populations.

### 3.3 Contemporary *Ne* Estimates

**FIGURE 5:**
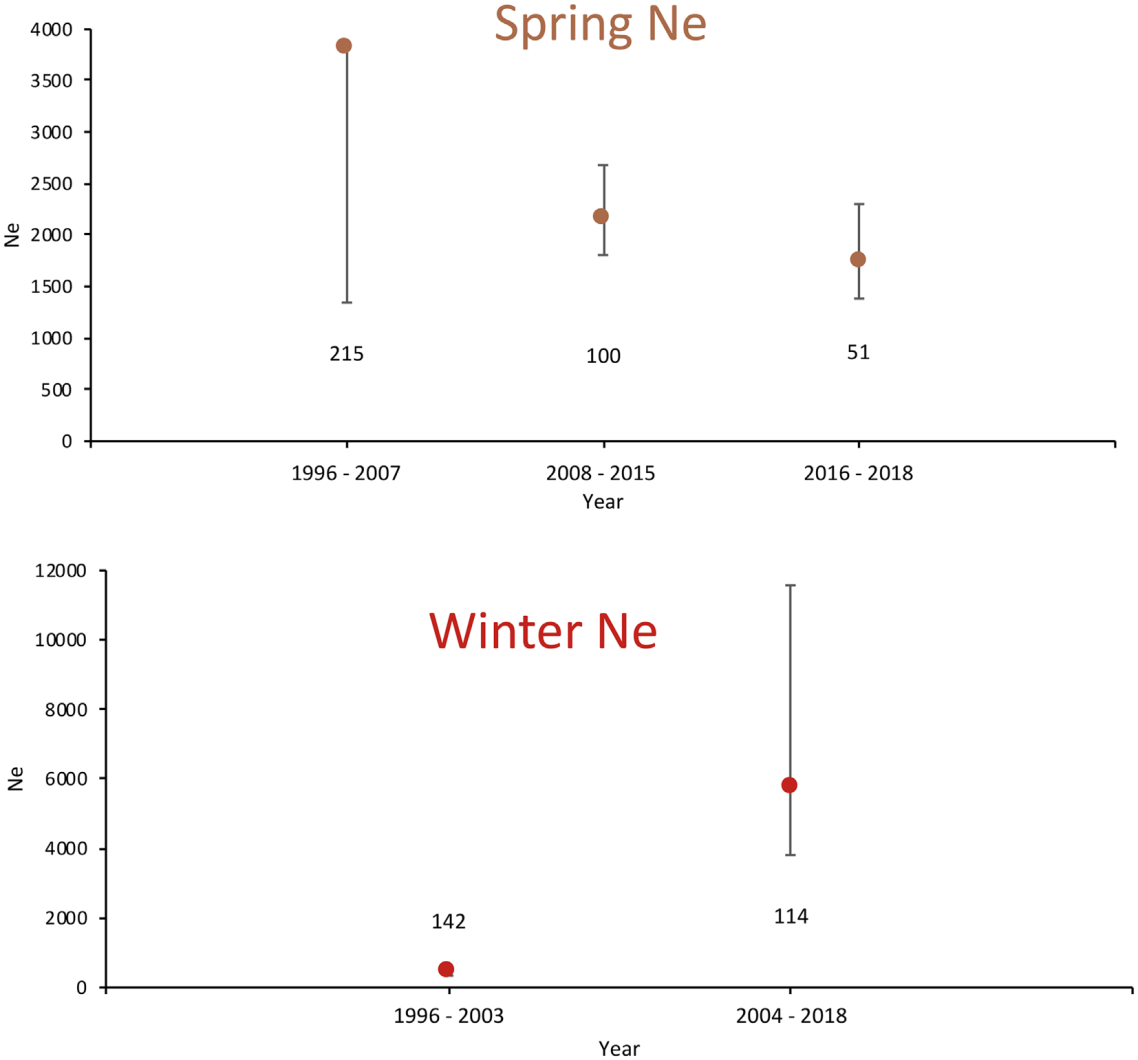
The *Ne* estimates calculated with NeEstimator for each population of the samples collected between 1996 and 2018 showed a decrease in *Ne* estimates over time for the Spring run populations. The upper confidence interval for the earliest spring samples was infinite. Note that the *y*‐axes bounds are not the same for each population. The number of samples is reported next to each data point.

Contemporary *Ne* values were estimated for Spring and Winter runs, grouped by collection years. A decline in *Ne* estimates over the last 20 years was detected in Spring run populations. The decrease in *Ne* estimates for Spring run CV Chinook salmon is consistent with observed decreases in abundances (Azat and Titus 2023). The Winter run population had a lower *Ne* value for the individuals collected from 1996–2003, followed by an increase from 2004 to 2018 (Figure 5). Within this study, the 1996–2003 Winter run population *Ne* estimates fell below the critical threshold of 1000. *Ne* below 1000 signals accelerated loss of genetic diversity and reduced adaptive capacity (Willi et al. 2022; Hoban et al. 2024; Franklin 1980; Jamieson and Allendorf 2012). In contrast to the historic *Ne* estimates, contemporary *Ne* estimates from the 1996–2018 samples have all dropped to below 10,000, underscoring a dramatic decline in Winter and Spring run Chinook salmon populations in the CV.

## 4 Discussion

In this study, we observed large changes in intraspecific genetic diversity over time for the ESA‐listed runs of Chinook salmon in the Central Valley. We observed substantial changes in historical *Ne*, suggesting that major declines in abundance and genetic diversity occurred between 1870 and when the samples were collected for this study. Additionally, results from Tajima's D neutrality tests revealed more subtle shifts in genetic diversity during the contemporary period, after the initial *Ne* reductions had taken place.

### 4.1 Historical Context of Chinook Salmon Declines in the Central Valley

The first historical written record of human impacts on CV Chinook salmon abundance described the increased effort required to fish for Chinook salmon in the Sacramento River as early as 1851 (Kirkpatrick 1860). Subsequent expansion of commercial fisheries and water‐development projects further altered population trajectories, although their effects differed among runs, contributing to the differential abundances observed today. Early commercial freshwater fisheries, which began in the 1850s, heavily targeted the Spring and Winter runs. Spring and Winter runs were highly valued because they arrived to freshwater spawning grounds earlier and in peak physical condition with high fatty reserves (Jordan and Gilbert 1887; Stone 1889; Jordan 1904; CFGC 1916; J. E. Skinner 1958; Fisher 1994).

The impacts of gold mining contributed to large declines in CV Chinook salmon. After gold mining peaked in California (1848–1855) half of the streams in the CV were no longer suitable habitat for spawning (CFC 1877; CFC 1880). In 1866, anglers also attributed declining salmon runs to mining sediment (Stone 1874). The timing of mining impacts corresponds with the onset of the precipitous decline in historical *Ne* estimates of the Spring run Butte Creek population and Winter run population in the 1860s–1870s (50 generations ago) (Figure 3). It is unclear which runs may have been most heavily impacted because there is a lack of accurate scientific and historical records dating before the earliest habitat disturbances.

Due to the noticeable decline in Chinook salmon in California, hatcheries were developed in 1872 to supplement declining populations. Some early hatchery practices, such as taking eggs out of the natural environment to establish the hatcheries and sending eggs to other regions to start new populations, were detrimental to the survival of the wild populations due to reduced fitness and genetic diversity of wild fish (Stone 1878, 1883, 1897; Clark 1929).

Early dam construction was the next blow to Chinook salmon populations in the CV. By 1884, salmon could no longer reach spawning grounds in the Stanislaus, Tuolumne, San Joaquin, and upper Sacramento Rivers due to dams blocking their routes (CFC 1884; Collins 1892; Yoshiyama et al. 1998). Subsequently, the canning industry peaked in 1885 and was eventually abolished in California in 1919 due to its detrimental impacts on salmon populations (CFC 1886; J. Skinner 1962; Yoshiyama et al. 1998). Impacts on Chinook salmon populations in the CV from early hatchery practices, early dam construction, and the canning industry correspond with the continued decline in historical *Ne* estimates of the Spring run Butte Creek population and Winter run population in the early 1900s (35–40 generations ago) (Figure 3).

### 4.2 Large Dam Construction Era and Accelerated Decline

The 1930s (25 generations ago) mark the point of steeper decline in historical *Ne* estimates (Figure 3). The 1930s is when construction of modern dams began, compounding the damages from early dams, the canning industry, and large‐scale fishing. Modern, impassible dams remain one of the most detrimental forces to healthy Chinook populations, have obstructed 95% of the historical CV Chinook salmon habitat (Schaller et al. 2014; Merz et al. 2024; NOAA 2020) and reduced water flow downstream, which is critical for salmon survival (Reynolds et al. 1993).

Most CV dams block historic Spring and Winter run spawning habitat, which is characterized by higher elevation streams cooled by snowmelt. These areas provided critical thermal refugia for adults holding through summer (CFC 1890; Yoshiyama et al. 2001). Spring run juveniles that remain in upstream headwaters before ocean migrations are key to cohort success during drought and high temperature years, enhancing overall population resilience and stability (Cordoleani et al. 2021). Devastatingly, the restriction of Spring and Winter run salmon to valley floor habitat during high temperatures leaves them particularly vulnerable to climate change as summers get warmer and droughts become more frequent (Crozier et al. 2019).

### 4.3 Interpretation and Limitations of Historical *Ne* Estimates

The very large historical *Ne* estimates inferred using GONE should not be interpreted as precise reconstructions of absolute historical population size, but are rather a relative value. These values indicate that historical *Ne* was extremely large relative to contemporary *Ne* values. GONE infers historical *Ne* from patterns of linkage disequilibrium across recombination distances and is sensitive to biological features common in salmonids, including overlapping generations, variance in reproductive success, and gene flow among neighboring populations. Accordingly, our historical *Ne* estimates should be interpreted as evidence for historically high genetic diversity and demographic buffering capacity rather than literal values. Importantly, these estimates are robust in their relative comparisons among populations and in the timing and magnitude of subsequent declines, which align closely with independent historical records of habitat loss, mining, dam construction, and industrial fisheries in California's Central Valley.

### 4.4 Contemporary Changes in Genetic Diversity

#### 4.4.1 Neutrality Tests

Our results revealed fine scale shifts in genetic diversity for both Spring and Winter run Chinook salmon in the Central Valley from 1996 to 2018. Both runs exhibited significant changes in nucleotide diversity (π), the relationships among θπ, θW, and θH (e.g., Fay & Wu's H), the SFS, and Tajima's D distributions, with declining means in recent years (Tables 1 and 2; Figure 4). These patterns indicate reduced genetic variation relative to neutral expectations (Tajima 1989) and right‐skewed Tajima's D distributions consistent with recent population contractions, though balancing selection or population structure may also contribute (Maruyama and Fuerst 1985). These genetic signals align with documented demographic declines in both runs (Azat and Titus 2023). Winter run Chinook salmon, listed as endangered in 1994, declined from over 100,000 fish in the 1960s to only a few hundred by the 1980s, with all historical populations extirpated (Lindley et al. 2004). Spring run Chinook, listed as threatened in 1996, similarly collapsed from many independent CV populations to just three wild populations (Mill, Deer, and Butte Creeks), limiting overall viability (Lindley et al. 2004). Although some Spring run populations rebounded briefly, a sharp decline in 2015 underscored ongoing vulnerability (Azat and Titus 2023).

#### 4.4.2 *Ne* Estimates

To contextualize recent declines, we estimated contemporary effective population size (*Ne*) across sampled years. Winter run *Ne* values from 1996 to 2003 fell between the critical thresholds of 100 and 1000, indicating accelerated loss of genetic diversity and reduced adaptive capacity, but not so low to be associated with risks of inbreeding, lower fitness, and increased extinction risk (Willi et al. 2022; Hoban et al. 2024; Franklin 1980; Jamieson and Allendorf 2012). This low *Ne* aligns with the sharp decline in Winter run abundance in the early 1990s (Figure 5; Azat and Titus 2023). *Ne* increased, rising above the critical threshold of 1000 for Winter run populations between 2004 and 2018, potentially influenced by managed cold water releases from the Shasta Reservoir and conservation supplementation from the Livingston Stone National Fish Hatchery (NOAA 2021).

Spring run populations showed a decline in *Ne* over the past two decades, consistent with observed reductions in abundance (Azat and Titus 2023). Historically the most abundant populations, Spring run populations have been reduced to naturally reproducing groups in only Butte, Mill, and Deer Creeks (Campbell and Moyle 1991; Fisher 1994; Williams 2006). All Spring run populations were above the critical threshold of 1000 *Ne* at all sample times.

Both runs once migrated to cool mountain streams to avoid summer valley heat, but widespread dam construction that continued until the 1970s eliminated much of this habitat, driving many populations to extinction (Table 3). Overall, *Ne* trends and genetic diversity statistics closely track contemporary declines in abundance driven by habitat loss, hatchery practices, and barriers to migration (Cordoleani et al. 2020; Goertler et al. 2020). Indigenous knowledge and scientific research alike underscore that salmon require access to cold, high elevation habitats for successful spawning and rearing (Quiñones et al. 2015; Atlas et al. 2021).

**TABLE 3:** List of dams and hatcheries in the temporal order of construction.

Year of construction	Project	River	Tributary to
1924	Exchequer Dam	Merced River	San Joaquin River
1924	Don Pedro Dam	Tuolumne River	San Joaquin River
1937	Friant Dam	San Joaquin River	San Joaquin River
1938	Shasta Dam	Sacramento River	Sacramento River
1941	Englebright Dam	Yuba River	Sacramento River
1941	Keswick Dam	Sacramento River	Sacramento River
1942	Coleman Hatchery	Battle Creek	Sacramento River
1947	Pine Flat Dam	Kings River	San Joaquin River
1958	Folsom Dam	American River	Sacramento River
1958	Nimbus Hatchery	American River	Sacramento River
1961	Oroville Dam	Feather River	Sacramento River
1963	New Hogan Dam	Calaveras River	San Joaquin River
1963	Comanche Dam	Mokelumne River	San Joaquin River
1963	Mokelumne Hatchery	Mokelumne River	San Joaquin River
1964	New Exchequer Dam	Merced River	San Joaquin River
1966	New Melones Dam	Stanislaus River	San Joaquin River
1967	New Don Pedro Dam	Tuolumne River	San Joaquin River
1967	Feather River Hatchery	Feather River	Sacramento River
1970	Merced River Hatchery	Merced River	San Joaquin River

*Note:* Each project is also categorized by the river impacted by the project and the major river to which the project river feeds into.

## 5 Management Implications and Conclusions

This study elucidates changes in genetic diversity and estimates effective population sizes over time for the ESA listed populations and runs of Chinook salmon in the CV. Observations of *Ne* estimates across time provide insight into the differences between historic and contemporary genetic diversity, helping to define what can be considered baseline *Ne* values. Historical Ne trajectories from this study reveal that dramatic population declines began over 50 generations ago, long before contemporary monitoring efforts. Historic *Ne* estimates were very large across runs, consistent with substantially greater historical genetic diversity than observed today, while contemporary estimates (1996–2018) have plummeted for all populations. This dramatic decline underscores the risk of shifting baseline syndrome, where managers may set recovery targets based on recent degraded conditions rather than pre‐exploitation population sizes (Guerrero‐Gatica et al. 2019; Thurow et al. 2020).

This study provides greater genetic context to help inform biologically meaningful recovery goals aligned with historical conditions (Rouyer et al. 2011; McClenachan et al. 2012; Schijns and Pauly 2022). Tracking *Ne* annually allows managers to detect genetic diversity loss more sensitively than abundance monitoring alone, enabling timely intervention decisions. *Ne* estimates are particularly useful for managers that need to make decisions for populations in dire condition, such as the Butte Creek Spring run population that experienced a pre‐spawning mortality event in 2021 resulting in lower than usual returns (Nichols 2022). When *Ne* falls below 500, as observed in Winter run populations from 1996 to 2003, populations face accelerated genetic diversity loss, reduced adaptive capacity, and elevated extinction risk (Franklin 1980; Jamieson and Allendorf 2012; Willi et al. 2022, Hoban et al. 2024).

When standardized historical abundance data is unavailable, our study demonstrates how contemporary samples can be used to estimate historical relative changes in genetic diversity and abundance. These methods can be informative in partnership with traditional ecological knowledge, and archeological, trade, and catch records (McClenachan et al. 2012; Pauly 1995; Pinnegar and Engelhard 2008; Rosenberg et al. 2005; Zeller and Pauly 2016; FiveCrows et al. 2023). Prior studies have reported associations between the incorporation of traditional ecological knowledge within Indigenous governance frameworks and conservation outcomes across a range of species (Atlas et al. 2021; Service et al. 2014; Frid et al. 2016; DeRoy et al. 2019). In this study, effective population size estimates were higher prior to colonization, corresponding to a period during which salmon populations were managed through Indigenous stewardship practices. These findings are consistent with previous work suggesting that such management systems were associated with long‐term population persistence (Atlas et al. 2021).

## Author Contributions

**E. E. Collins:** formal analysis (equal), investigation (equal), methodology (equal), validation (equal), visualization (equal), writing – original draft (equal), writing – review and editing (equal). **T. Q. Thompson:** conceptualization (equal), data curation (equal), investigation (equal), methodology (equal), validation (equal), writing – review and editing (equal). **P. Goertler:** conceptualization (equal), data curation (equal), investigation (equal), project administration (equal), resources (equal), writing – review and editing (equal). **M. R. Baerwald:** conceptualization (equal), data curation (equal), investigation (equal), project administration (equal), resources (equal), writing – review and editing (equal). **M. H. Meek:** conceptualization (equal), funding acquisition (equal), investigation (equal), methodology (equal), project administration (equal), resources (equal), supervision (equal), validation (equal), visualization (equal), writing – original draft (equal), writing – review and editing (equal).

## Funding

Funding for this work was provided by a grant from the State of California Delta Stewardship Council (Agreement no. 18209).

## Ethics Statement

This study used archived fish tissue samples transferred from the California Fish Tissue Archive under a Transfer of Possession–Chain of Custody agreement. No live animals were captured, handled, or euthanized as part of this research. The authors' work was restricted to genetic analyses of previously collected specimens.

## Conflicts of Interest

The authors declare no conflicts of interest.

## Supporting information


**Data S1:** Supporting Information.

## Data Availability

Genotype likelihood data are available in Dryad. Dataset link for reviewers https://doi.org/10.5061/dryad.tht76hfdr.

## References

[ece374176-bib-0001] Ali, O. A. , S. M. O'Rourke , S. J. Amish , et al. 2016. “RAD Capture (Rapture): Flexible and Efficient Sequence‐Based Genotyping.” Genetics 202, no. 2: 389–400. 26715661 10.1534/genetics.115.183665 PMC4788223

[ece374176-bib-0002] Allendorf, F. , N. Ryman , and F. Utter . 1987. Genetics and Fishery Management: Past, Present, and Future, 1–19. Washington Sea Grant Publications/University of Washington Press.

[ece374176-bib-0003] Atlas, W. I. , N. C. Ban , J. W. Moore , et al. 2021. “Indigenous Systems of Management for Culturally and Ecologically Resilient Pacific Salmon (*Oncorhynchus spp*.) Fisheries.” Bioscience 71, no. 2: 186–204. 33613129 10.1093/biosci/biaa144 PMC7882363

[ece374176-bib-0004] Atlas, W. I. , M. R. Sloat , W. H. Satterthwaite , et al. 2023. “Trends in Chinook Salmon Spawner Abundance and Total Run Size Highlight Linkages Between Life History, Geography and Decline.” Fish and Fisheries 24, no. 4: 595–617.

[ece374176-bib-0005] Azat, J. , and R. Titus . 2023. California Central Valley Chinook Escapement Database Report “GrandTab 2023.06.26”, 1–29. California Department of Fish and Wildlife Fisheries Branch.

[ece374176-bib-0008] Beebee, T. J. C. 2009. “A Comparison of Single‐Sample Effective Size Estimators Using Empirical Toad ( *Bufo calamita* ) Population Data: Genetic Compensation and Population Size‐Genetic Diversity Correlations.” Molecular Ecology 18, no. 23: 4790–4797. 19863715 10.1111/j.1365-294X.2009.04398.x

[ece374176-bib-0009] Berkeley, S. A. , M. A. Hixon , R. J. Larson , and M. S. Love . 2004. “Fisheries Sustainability via Protection of Age Structure and Spatial Distribution of Fish Populations.” Fisheries 29, no. 8: 23–32.

[ece374176-bib-0010] Brandes, P. L. , and J. S. McLain . 2001. “Juvenile Chinook Salmon Abundance, Distribution, and Survival in the Sacramento–San Joaquin Estuary.” In Fish Bulletin 179. Contributions to the Biology of the Central Valley Salmonids, Vol. 2, edited by R. L. Brown , 39–136. California Department of Fish and Game. https://escholarship.org/uc/item/6sd4z5b2.

[ece374176-bib-0146] Brown, C. J. , and R. Trebilco . 2014. “Unintended Cultivation, Shifting Baselines, and Conflict Between Objectives for Fisheries and Conservation.” Conservation Biology 28, no. 3: 677–688. 24665891 10.1111/cobi.12267

[ece374176-bib-0011] Campbell, E. A. , and P. B. Moyle . 1991. “Historical and Recent Population Sizes of Spring‐Run Chinook salmon in California.” In *Proceedings of the 1990 Northeast Pacific Chinook and Coho Salmon Workshop*, 155–216. American Fisheries Society, Humboldt State University.

[ece374176-bib-0012] Carney, M. , S. Tushingham , T. McLaughlin , and G. J. d'Alpoim . 2021. “Harvesting Strategies as Evidence for 4000 Years of Camas ( *Camassia quamash* ) Management in the North American Columbia Plateau.” Royal Society Open Science 8, no. 4: 202213. 33996124 10.1098/rsos.202213 PMC8059633

[ece374176-bib-0013] CFC . 1877. Report of the Commissioners of Fisheries of the State of California, for the Years 1876 and 1877. CFC.

[ece374176-bib-0014] CFC . 1880. Report of the Commissioners of Fisheries of the State of California, for the Year of 1880. CFC.

[ece374176-bib-0015] CFC . 1884. Report of the Commissioners of Fisheries of the State of California, for the Years 1883–1884. CFC.

[ece374176-bib-0016] CFC . 1886. Report of the Commissioners of Fisheries of the State of California, for 1885–1886. CFC.

[ece374176-bib-0017] CFC . 1890. Report of the Commissioners of Fisheries of the State of California, for the Years 1888–1890. CFC.

[ece374176-bib-0019] CFGC (California Fish and Game Commission) . 1916. Report of Department of Commercial Fisheries. CFGC.

[ece374176-bib-0020] Christensen, K. A. , J. S. Leong , D. Sakhrani , et al. 2018. “Chinook Salmon ( *Oncorhynchus tshawytscha* ) Genome and Transcriptome.” PLoS One 13, no. 4: e0195461. 29621340 10.1371/journal.pone.0195461 PMC5886536

[ece374176-bib-0021] Clark, G. H. 1929. Sacramento–San Joaquin Salmon ( *Oncorhynchus tshawytscha* ) Fishery of California. Vol. 17. California Department of Fish and Game.

[ece374176-bib-0151] Collins, J. W. 1892. Report on the Fisheries of the Pacific Coast of the United States . Report of the Commissioner for 1888, Appendix 1, 3–269. United States Commission of Fish and Fisheries.

[ece374176-bib-0023] Cordoleani, F. , C. C. Phillis , A. M. Sturrock , et al. 2021. “Threatened Salmon Rely on a Rare Life History Strategy in a Warming Landscape.” Nature Climate Change 11, no. 11: 982–988.

[ece374176-bib-0024] Cordoleani, F. , W. H. Satterthwaite , M. E. Daniels , and M. R. Johnson . 2020. “Using Life‐Cycle Models to Identify Monitoring Gaps for Central Valley Spring‐Run Chinook Salmon.” San Francisco Estuary and Watershed Science 18, no. 4: 1–30.

[ece374176-bib-0025] Crozier, L. G. , M. M. McClure , T. Beechie , et al. 2019. “Climate Vulnerability Assessment for Pacific Salmon and Steelhead in the California Current Large Marine Ecosystem.” PLoS One 14, no. 7: e0217711. 31339895 10.1371/journal.pone.0217711 PMC6655584

[ece374176-bib-0026] Danecek, P. , J. K. Bonfield , J. Liddle , et al. 2021. “Twelve Years of SAMtools and BCFtools.” GigaScience 10, no. 2: egiab008. 10.1093/gigascience/giab008 PMC7931819 33590861

[ece374176-bib-0027] DeRoy, B. , C. T. Darimont , and C. N. Service . 2019. “Biocultural Indicators to Support Locally Led Environmental Management and Monitoring.” Ecology and Society 24, no. 4: 1–14.31798644

[ece374176-bib-0028] Dettinger, M. 2011. “Climate Change, Atmospheric Rivers, and Floods in California–a Multimodel Analysis of Storm Frequency and Magnitude Changes.” Journal of the American Water Resources Association 47, no. 3: 514–523.

[ece374176-bib-0029] Do, C. , R. S. Waples , D. Peel , G. M. Macbeth , B. J. Tillett , and J. R. Ovenden . 2014. “NeEstimator V2: Re‐Implementation of Software for the Estimation of Contemporary Effective Population Size *(Ne)* From Genetic Data.” Molecular Ecology Resources 14: 209–214. 23992227 10.1111/1755-0998.12157

[ece374176-bib-0150] Exposito‐Alonso, M. , T. R. Booker , L. Czech , et al. 2022. “Genetic Diversity Loss in the Anthropocene.” Science 377, no. 6613: 1431–1435. 36137047 10.1126/science.abn5642

[ece374176-bib-0030] FAO (Food and Agriculture Organization) . 2020. The State of World Fisheries and Aquaculture 2020. Sustainability in Action. Food and Agriculture Organization of the United Nations.

[ece374176-bib-0031] Fisher, A. C. , W. M. Hanemann , and A. G. Keeler . 1991. “Integrating Fishery and Water Resource Management: A Biological Model of a California Salmon Fishery.” Journal of Environmental Economics and Management 20, no. 3: 234–261.

[ece374176-bib-0032] Fisher, F. W. 1994. “Past and Present Status of Central Valley Chinook Salmon.” Conservation Biology 8: 870–873.

[ece374176-bib-0033] FiveCrows, J. , A. DeCoteau , J. Hess , D. Hatch , and S. Narum . 2023. “Sharing Biological Information Across Generations: Parallels Between Indigenous Knowledge and Genetics for Fisheries Recovery in the Columbia River Basin.” Molecular Ecology Resources 25, no. 2: e13815.

[ece374176-bib-0034] Frankham, R. , C. J. A. Bradshaw , and B. W. Brook . 2014. “Genetics in Conservation Management: Revised Recommendations for the 50/500 Rules, Red List Criteria and Population Viability Analyses.” Biological Conservation 170: 56–63. 10.1016/j.biocon.2013.12.036.

[ece374176-bib-0035] Franklin, I. R. 1980. “Evolutionary Change in Small Populations.” In Conservation Biology: An Evolutionary‐Ecological Perspective, 395. Sinauer Associates.

[ece374176-bib-0036] Frid, A. , M. McGreer , and A. Stevenson . 2016. “Rapid Recovery of Dungeness Crab Within Spatial Fishery Closures Declared Under Indigenous Law in British Columbia.” Global Ecology and Conservation 6: 48–57.

[ece374176-bib-0037] Froese, R. , D. Zeller , K. Kleisner , and D. Pauly . 2012. “What Catch Data Can Tell Us About the Status of Global Fisheries.” Marine Biology 159: 1283–1292.

[ece374176-bib-0039] Goertler, P. , F. Cordoleani , J. Notch , R. Johnson , and G. Singer . 2020. Spring‐Run Workshop Factsheet Life history variation in Central Valley spring‐run Chinook. 10.13140/RG.2.2.21153.53600.

[ece374176-bib-0040] Guerrero‐Gatica, M. , E. Aliste , and J. A. Simonetti . 2019. “Shifting Gears for the Use of the Shifting Baseline Syndrome in Ecological Restoration.” Sustainability 11, no. 5: 1458.

[ece374176-bib-0042] Hayes, B. J. , P. M. Visscher , H. C. McPartlan , and M. E. Goddard . 2003. “Novel Multilocus Measure of Linkage Disequilibrium to Estimate Past Effective Population Size.” Genome Research 13, no. 4: 635–643. 12654718 10.1101/gr.387103 PMC430161

[ece374176-bib-0043] Hilborn, R. , T. P. Quinn , D. E. Schindler , and D. E. Rogers . 2003. “Biocomplexity and Fisheries Sustainability.” Proceedings of the National Academy of Sciences 100: 6564–6568. 10.1073/pnas.1037274100. PMC164486 12743372

[ece374176-bib-0044] Hill, W. G. 1981. “Estimation of Effective Population Size From Data on Linkage Disequilibrium.” Genetics Research 38, no. 3: 209–216.

[ece374176-bib-0045] Hoban, S. , F. I. Archer , L. D. Bertola , et al. 2022. “Global Genetic Diversity Status and Trends: Towards a Suite of Essential Biodiversity Variables (EBVs) for Genetic Composition.” Biological Reviews 97, no. 4: 1511–1538. 35415952 10.1111/brv.12852 PMC9545166

[ece374176-bib-0149] Hoban, S. , M. W. Bruford , W. C. Funk , et al. 2021. “Global Commitments to Conserving and Monitoring Genetic Diversity are Now Necessary and Feasible.” Bioscience 71, no. 9: 964–976. 34475806 10.1093/biosci/biab054 PMC8407967

[ece374176-bib-0147] Hoban, S. , J. M. da Silva , A. Hughes , et al. 2024. “Too Simple, Too Complex, or Just Right? Advantages, Challenges, and Guidance for Indicators of Genetic Diversity.” Bioscience 74, no. 4: 269–280.

[ece374176-bib-0046] Hoban, S. , J. M. da Silva , A. Mastretta‐Yanes , et al. 2023. “Monitoring Status and Trends in Genetic Diversity for the Convention on Biological Diversity: An Ongoing Assessment of Genetic Indicators in Nine Countries.” Conservation Letters 16, no. 3: e12953.

[ece374176-bib-0047] Hu, Y. , H. Shang , H. Tong , et al. 2009. “Stable Isotope Dietary Analysis of the Tianyuan 1 Early Modern Human.” Proceedings of the National Academy of Sciences 106, no. 27: 10971–10974. 10.1073/pnas.0904826106 PMC2706269 19581579

[ece374176-bib-0049] Jackson, J. B. , K. E. Alexander , and E. Sala . 2011. Shifting Baselines: The Past and Future of Ocean Fisheries. Island Press.

[ece374176-bib-0050] Jackson, J. B. , M. X. Kirby , W. H. Berger , et al. 2001. “Historical Overfishing and the Recent Collapse of Coastal Ecosystems.” Science 293, no. 5530: 629–637. 11474098 10.1126/science.1059199

[ece374176-bib-0051] Jamieson, I. G. , and F. W. Allendorf . 2012. “How Does the 50/500 Rule Apply to MVPs?” Trends in Ecology & Evolution 27, no. 10: 578–584. 10.1016/j.tree.2012.07.001. 22868005

[ece374176-bib-0052] Jennings, S. , and J. L. Blanchard . 2004. “Fish Abundance With no Fishing: Predictions Based on Macroecological Theory.” Journal of Animal Ecology 73: 632–642.

[ece374176-bib-0055] Jordan, D. S. 1904. “Pacific Species of Salmon and Trout.” In Eighteenth Biennial Report of the State Board of Fish Commissioners of the State of California, 75–97. State of California.

[ece374176-bib-0056] Jordan, D. S. , and C. H. Gilbert . 1887. “The Salmon Fishing and Canning Interests of the Pacific Coast.” In The Fisheries and Fishery Industries of the United States, Section V, History and Methods of Fishing, vol. 1, 729–753. US Government Printing Office in Washington D.C.

[ece374176-bib-0057] Kalinowski, S. T. , and R. S. Waples . 2002. “Relationship of Effective to Census Size in Fluctuating Populations.” Conservation Biology 16: 129–136. 10.1046/j.1523-1739.2002.00134.x. 35701960

[ece374176-bib-0059] Kirkpatrick, C. A. 1860. “Salmon Fishery on the Sacramento River.” Hutchings' California Magazine 4, no. 12: 529–534.

[ece374176-bib-0060] Kittinger, J. N. , L. McClenachan , K. B. Gedan , and L. K. Blight . 2015. Marine Historical Ecology in Conservation: Applying the Past to Manage for the Future, 1–118. University of California Press.

[ece374176-bib-0061] Korneliussen, T. S. , A. Albrechtsen , and R. Nielsen . 2014. “ANGSD: Analysis of Next Generation Sequencing Data.” BMC Bioinformatics 15, no. 1: 1–13. 25420514 10.1186/s12859-014-0356-4 PMC4248462

[ece374176-bib-0062] Layton, K. K. S. , P. V. R. Snelgrove , J. B. Dempson , et al. 2021. “Genomic Evidence of Past and Future Climate‐Linked Loss in a Migratory Arctic Fish.” Nature Climate Change 11, no. 2: 158–165.

[ece374176-bib-0148] Leigh, D. M. , A. P. Hendry , E. Vázquez‐Domínguez , and V. L. Friesen . 2019. “Estimated Six Per Cent Loss of Genetic Variation in Wild Populations Since the Industrial Revolution.” Evolutionary Applications 12, no. 8: 1505–1512. 31462910 10.1111/eva.12810 PMC6708419

[ece374176-bib-0063] Lindley, S. T. , R. Schick , B. P. May , et al. 2004. Population Structure of Threatened and Endangered Chinook Salmon ESUs in California's Central Valley Basin, 1–38. NOAA Technical Memo.

[ece374176-bib-0064] Lotze, H. K. , and B. Worm . 2009. “Historical Baselines for Large Marine Animals.” Trends in Ecology & Evolution 24, no. 5: 254–262. 19251340 10.1016/j.tree.2008.12.004

[ece374176-bib-0065] Luikart, G. , and J. M. Cornuet . 1999. “Estimating the Effective Number of Breeders From Heterozygote Excess in Progeny.” Genetics 151, no. 3: 1211–1216. 10049936 10.1093/genetics/151.3.1211 PMC1460520

[ece374176-bib-0066] Mamoozadeh, N. R. , M. J. Wade , B. N. Reid , et al. 2025. “A Practical Introduction to Effective Population Size for Fisheries Management.” Transactions of the American Fisheries Society 154, no. 4: 352–371.

[ece374176-bib-0068] Maruyama, T. , and P. A. Fuerst . 1985. “Population Bottlenecks and Nonequilibrium Models in Population Genetics. II. Number of Alleles in a Small Population That Was Formed by a Recent Bottleneck.” Genetics 111, no. 3: 675–689. 4054612 10.1093/genetics/111.3.675 PMC1202664

[ece374176-bib-0069] Matsaw, S. L. 2020. Teachings From the Land of My Ancestors: Knowing Places as a Gatherer, Hunter, Fisher and Ecologist, 73–85. Springer.

[ece374176-bib-0070] May, R. M. 1994. “Biological Diversity: Differences Between Land and Sea.” Philosophical Transactions of the Royal Society of London. Series B, Biological Sciences 343: 105–111.

[ece374176-bib-0071] McClenachan, L. , F. Ferretti , and J. K. Baum . 2012. “From Archives to Conservation: Why Historical Data Are Needed to Set Baselines for Marine Animals and Ecosystems.” Conservation Letters 5, no. 5: 349–359.

[ece374176-bib-0072] Meek, M. H. , M. R. Baerwald , M. R. Stephens , et al. 2016. “Sequencing Improves Our Ability to Study Threatened Migratory Species: Genetic Population Assignment in California's Central Valley Chinook Salmon.” Ecology and Evolution 6: 7706–7716. 10.1002/ece3.2493. 30128122 PMC6093154

[ece374176-bib-0073] Meek, M. H. , M. R. Stephens , A. Goodbla , B. May , and M. R. Baerwald . 2020. “Identifying Hidden Biocomplexity and Genomic Diversity in Chinook Salmon, an Imperiled Species With a History of Anthropogenic Influence.” Canadian Journal of Fisheries and Aquatic Sciences 77, no. 3: 534–547.

[ece374176-bib-0074] Merz, J. E. , R. A. Brown , K. Sellheim , and S. C. Zeug . 2024. “Disruption of Natural Disturbance Regime Decouples Habitat and Life Stage in a Keystone Species.” Ecosphere 15, no. 10: e70017.

[ece374176-bib-0075] Messer, P. W. , S. P. Ellner , and N. G. Hairston . 2016. “Can Population Genetics Adapt to Rapid Evolution?” Trends in Genetics 32, no. 7: 408–418. 27185237 10.1016/j.tig.2016.04.005

[ece374176-bib-0076] Mimura, M. , T. Yahara , D. P. Faith , et al. 2017. “Understanding and Monitoring the Consequences of Human Impacts on Intraspecific Variation.” Evolutionary Applications 10, no. 2: 121–139. 28127389 10.1111/eva.12436 PMC5253428

[ece374176-bib-0077] Mitchell, M. 1998. An Introduction to Genetic Algorithms, 1–186. MIT Press.

[ece374176-bib-0078] Nadachowska‐Brzyska, K. , M. Konczal , and W. Babik . 2022. “Navigating the Temporal Continuum of Effective Population Size.” Methods in Ecology and Evolution 13, no. 1: 22–41.

[ece374176-bib-0079] Narum, S. R. , A. Di Genova , S. Micheletti , and A. Maass . 2018. “Genomic Variation Underlying Complex Life‐History Traits Revealed by Genome Sequencing in Chinook Salmon.” Proceedings of the Royal Society B: Biological Sciences 285: 20180935. 10.1098/rspb.2018.0935 PMC6083255 30051839

[ece374176-bib-0081] Nelson, P. A. , M. Baerwald , O. T. Burgess , et al. 2022. “Considerations for the Development of a Juvenile Production Estimate for Central Valley Spring‐Run Chinook Salmon.” San Francisco Estuary and Watershed Science 20, no. 2: 1–23.

[ece374176-bib-0082] Nichols, J. 2022. Butte Creek Spring‐Run Chinook Salmon Adult Monitoring Annual Report 2021. State of California, Natural Resources Agency Department of Fish and Wildlife.

[ece374176-bib-0083] NOAA . 2005. Endangered and Threatened Species: Final Listing Determinations for 16 ESUs of West Coast Salmon, and Final 4(d) Protective Regulations for Threatened Salmonid ESUs, 70 Fed. Reg. 37160‐37204.

[ece374176-bib-0084] NOAA . 2020. Salmon and Steelhead Habitat Loss in the Central Valley. https://noaa.maps.arcgis.com/apps/MapJournal/index.html?appid=ceebefd9685143daa5bf30d5a7e0c7fa.

[ece374176-bib-0085] NOAA . 2021. Species in the Spotlight Priority Actions 2021–2025. https://media.fisheries.noaa.gov/2021‐04/SIS%20Action%20Plan%202021_SacWinterRunChinook_FINAL%20508.pdf.

[ece374176-bib-0086] NOAA (National Oceanic and Atmospheric Administration) . 1994. Endangered and Threatened Species; Status of Sacramento River Winter‐Run Chinook Salmon; Final Rule, 59 Fed. Reg. 440–450.

[ece374176-bib-0087] Nomura, T. 2008. “Estimation of Effective Number of Breeders From Molecular Coancestry of Single Cohort Sample.” Evolutionary Applications 1, no. 3: 462–474. 25567728 10.1111/j.1752-4571.2008.00015.x PMC3352377

[ece374176-bib-0088] O'Leary, S. J. , T. Q. Thompson , and M. H. Meek . 2024. “Every Cog and Wheel: Identifying Biocomplexity at the Genomic and Phenotypic Level in a Population Complex of Chinook Salmon.” San Francisco Estuary and Watershed Science 22, no. 4.

[ece374176-bib-0090] Pauls, S. U. , C. Nowak , M. Bálint , and M. Pfenninger . 2013. “The Impact of Global Climate Change on Genetic Diversity Within Populations and Species.” Molecular Ecology 22, no. 4: 925–946. 23279006 10.1111/mec.12152

[ece374176-bib-0091] Pauly, D. 1995. “Anecdotes and the Shifting Baseline Syndrome of Fisheries.” Trends in Ecology & Evolution 10, no. 10: 430. 21237093 10.1016/s0169-5347(00)89171-5

[ece374176-bib-0092] Pauly, D. , V. Christensen , S. Guénette , et al. 2002. “Towards Sustainability in World Fisheries.” Nature 418, no. 6898: 689–695. 12167876 10.1038/nature01017

[ece374176-bib-0093] Petrolo, E. , J. Boomer , J. O'Hare , K. Bilgmann , and A. Stow . 2021. “Stock Structure and Effective Population Size of the Commercially Exploited Gummy Shark *Mustelus antarcticus* .” Marine Ecology Progress Series 678: 109–124.

[ece374176-bib-0094] Phillis, C. C. , A. M. Sturrock , R. C. Johnson , and P. K. Weber . 2018. “Endangered Winter‐Run Chinook Salmon Rely on Diverse Rearing Habitats in a Highly Altered Landscape.” Biological Conservation 217: 358–362.

[ece374176-bib-0095] Pinnegar, J. K. , and G. H. Engelhard . 2008. “The ‘Shifting Baseline’ Phenomenon: A Global Perspective.” Reviews in Fish Biology and Fisheries 18: 1–16.

[ece374176-bib-0096] Pitcher, T. J. 2001. “Fisheries Managed to Rebuild Ecosystems? Reconstructing the Past to Salvage the Future.” Ecological Applications 11, no. 2: 601–617.

[ece374176-bib-0097] Préfontaine, R. 2009. Shifting Baselines in Marine Fish Assessments: Implications for Perception of Management and Conservation Status. Honours Bachelor Thesis. Dalhousie University.

[ece374176-bib-0098] Prince, D. J. , S. M. O'Rourke , T. Q. Thompson , et al. 2017. “The Evolutionary Basis of Premature Migration in Pacific Salmon Highlights the Utility of Genomics for Informing Conservation.” Science Advances 3, no. 8: e1603198. 28835916 10.1126/sciadv.1603198 PMC5559211

[ece374176-bib-0099] Pudovkin, A. I. , D. V. Zaykin , and D. Hedgecock . 1996. “On the Potential for Estimating the Effective Number of Breeders From Heterozygote‐Excess in Progeny.” Genetics 144, no. 1: 383–387. 8878701 10.1093/genetics/144.1.383 PMC1207510

[ece374176-bib-0100] Pudovkin, A. I. , O. L. Zhdanova , and D. Hedgecock . 2010. “Sampling Properties of the Heterozygote‐Excess Estimator of the Effective Number of Breeders.” Conservation Genetics 11, no. 3: 759–771.

[ece374176-bib-0101] Pyper, B. , T. Garrison , S. Cramer , P. L. Brandes , D. P. Jacobson , and M. A. Banks . 2013. Absolute Abundance Estimates of Juvenile Spring‐Run and Winter‐Run Chinook Salmon at Chipps Island. Funded by the Delta Stewardship Council Delta Science Program (previously CALFED Bay‐Delta Program) Grant Agreement Number 1049. https://sciencetracker.deltacouncil.ca.gov/sites/default/files/Final%20Chipps_DNA_Abundance_Report_7_2_13_2_2.pdf.

[ece374176-bib-0102] Quaempts, E. J. , K. L. Jones , S. J. O'Daniel , T. J. Beechie , and G. C. Poole . 2018. “Aligning Environmental Management With Ecosystem Resilience: A First Foods Example From the Confederated Tribes of the Umatilla Indian Reservation, Oregon, USA.” Ecology and Society 23, no. 2: 29.

[ece374176-bib-0103] Quinn, T. P. 2021. “Differential Migration in Pacific Salmon and Trout: Patterns and Hypotheses.” Animal Migration 8: 1–18.

[ece374176-bib-0105] Quiñones, R. M. , T. E. Grantham , B. N. Harvey , et al. 2015. “Dam Removal and Anadromous Salmonid (Oncorhynchus spp.) Conservation in California.” Reviews in Fish Biology and Fisheries 25, no. 1: 195–215.

[ece374176-bib-0107] Reynolds, F. L. , T. J. Mills , R. Benthin , and A. Low . 1993. Restoring Central Valley Streams: A Plan of Sacramento. Action. California Department of Fish and Game.

[ece374176-bib-0108] Robuchon, M. , J. da Silva , G. Dubois , et al. 2023. “Conserving Species' Evolutionary Potential and History: Opportunities Under the Kunming–Montreal Global Biodiversity Framework.” Conservation Science and Practice 5: e12929.

[ece374176-bib-0109] Rosenberg, A. A. , W. J. Bolster , K. E. Alexander , W. B. Leavenworth , A. B. Cooper , and M. G. McKenzie . 2005. “The History of Ocean Resources: Modeling Cod Biomass Using Historical Records.” Frontiers in Ecology and the Environment 3, no. 2: 78–84.

[ece374176-bib-0110] Rouyer, T. , G. Ottersen , J. M. Durant , et al. 2011. “Shifting Dynamic Forces in Fish Stock Fluctuations Triggered by Age Truncation?” Global Change Biology 17, no. 10: 3046–3057.

[ece374176-bib-0111] Santiago, E. , I. Novo , A. F. Pardiñas , M. Saura , J. Wang , and A. Caballero . 2020. “Recent Demographic History Inferred by High‐Resolution Analysis of Linkage Disequilibrium.” Molecular Biology and Evolution 37, no. 12: 3642–3653. 32642779 10.1093/molbev/msaa169

[ece374176-bib-0112] Schaller, H. A. , C. E. Petrosky , and E. S. Tinus . 2014. “Evaluating River Management During Seaward Migration to Recover Columbia River Stream‐Type Chinook Salmon Considering the Variation in Marine Conditions.” Canadian Journal of Fisheries and Aquatic Sciences 71, no. 2: 259–271.

[ece374176-bib-0113] Schijns, R. , and D. Pauly . 2022. “Management Implications of Shifting Baselines in Fish Stock Assessments.” Fisheries Management and Ecology 29, no. 2: 183–195.

[ece374176-bib-0114] Schindler, D. E. , R. Hilborn , B. Chasco , et al. 2010. “Population Diversity and the Portfolio Effect in an Exploited Species.” Nature 465: 609–612. 10.1038/nature09060. 20520713

[ece374176-bib-0115] Schmidt, C. , S. Hoban , and W. Jetz . 2023. “Conservation Macrogenetics: Harnessing Genetic Data to Meet Conservation Commitments.” Trends in Genetics 39, no. 11: 816–829. 37648576 10.1016/j.tig.2023.08.002

[ece374176-bib-0116] Service, C. N. , M. S. Adams , K. A. Artelle , P. Paquet , L. V. Grant , and C. T. Darimont . 2014. “Indigenous Knowledge and Science Unite to Reveal Spatial and Temporal Dimensions of Distributional Shift in Wildlife of Conservation Concern.” PLoS One 9, no. 7: e101595. 25054635 10.1371/journal.pone.0101595 PMC4108310

[ece374176-bib-0117] Shrimpton, J. M. , and D. D. Heath . 2003. “Census vs. Effective Population Size in Chinook Salmon: Large‐and Small‐Scale Environmental Perturbation Effects.” Molecular Ecology 12, no. 10: 2571–2583. 12969462 10.1046/j.1365-294x.2003.01932.x

[ece374176-bib-0118] Skinner, J. 1962. A Historical Review of the Fish and Wildlife Resources of the San Francisco Bay Area, 57–65. California Department of Fish and Game, 1.

[ece374176-bib-0119] Skinner, J. E. 1958. Some Observations Regarding the King Salmon Runs of the Central Valley. California Department of Fish and Game, 1.

[ece374176-bib-0120] Stone, L. 1874. Report of Operations During 1872 at the United States Salmon‐Hatching Establishment on the M'cloud River and on the California Salmonidae Generally, 168–215. U.S. Commission of Fish and Fisheries, Report of the Commissioner.

[ece374176-bib-0121] Stone, L. 1878. Operations on the McCloud River in Salmon Breeding, 921–933. U.S. Commission of Fish and Fisheries, Report of the Commissioner, 10.

[ece374176-bib-0122] Stone, L. 1883. Account of Operations at the McCloud River Fish‐Breeding Stations of the United States Fish Commission, 217–236. U.S. Fish Commission Bulletin, 2.

[ece374176-bib-0123] Stone, L. 1889. “Best Season for Packing Salmon on the Pacific Coast.” U.S. Fish Commission Bulletin 7: 65–66.

[ece374176-bib-0124] Stone, L. 1897. “The Artificial Propagation of Salmon on the Pacific Coast of the United States, With Notes on the Natural History of the Quinnat Salmon.” U.S. Fish Commission Bulletin 16: 203–235.

[ece374176-bib-0125] Swain, D. L. , B. Langenbrunner , J. D. Neelin , and A. Hall . 2018. “Increasing Precipitation Volatility in Twenty‐First‐Century California.” Nature Climate Change 8, no. 5: 427–433.

[ece374176-bib-0126] Tajima, F. 1989. “Statistical Method for Testing the Neutral Mutation Hypothesis by DNA Polymorphism.” Genetics 123, no. 3: 585–595. 2513255 10.1093/genetics/123.3.585 PMC1203831

[ece374176-bib-0127] Thompson, N. F. , E. C. Anderson , A. J. Clemento , et al. 2020. “A Complex Phenotype in Salmon Controlled by a Simple Change in Migratory Timing.” Science 370, no. 6516: 609–613. 33122386 10.1126/science.aba9059

[ece374176-bib-0128] Thompson, T. Q. , S. J. O'Leary , S. O'Rourke , P. Goertler , M. R. Baerwald , and M. H. Meek . 2024. “Genomics and 20 Years of Sampling Reveal Phenotypic Differences Between Subpopulations of Outmigrating Central Valley Chinook Salmon.” Evolutionary Applications 17, no. 6: e13705. 10.1111/eva.13705. 38832083 PMC11146144

[ece374176-bib-0152] Thurow, R. F. , T. Copeland , and B. N. Oldemeyer . 2020. “Wild Salmon and the Shifting Baseline Syndrome: Application of Archival and Contemporary Redd Counts to Estimate Historical Chinook Salmon (*Oncorhynchus tshawytscha*) Production Potential in the Central Idaho Wilderness.” Canadian Journal of Fisheries and Aquatic Sciences 77, no. 4: 651–665.

[ece374176-bib-0130] Vasimuddin, M. , S. Misra , H. Li , and S. Aluru . 2019. Efficient Architecture‐Aware Acceleration of BWA‐MEM for Multicore Systems, 314–324. IEEE Parallel and Distributed Processing Symposium (IPDPS). 10.1109/IPDPS.2019.00041.

[ece374176-bib-0131] Wang, J. 2009. “A New Method for Estimating Effective Population Sizes From a Single Sample of Multilocus Genotypes.” Molecular Ecology 18, no. 10: 2148–2164. 19389175 10.1111/j.1365-294X.2009.04175.x

[ece374176-bib-0132] Wang, J. 2016. “A Comparison of Single‐Sample Estimators of Effective Population Sizes From Genetic Marker Data.” Molecular Ecology 25, no. 19: 4692–4711. 27288989 10.1111/mec.13725

[ece374176-bib-0133] Wang, J. 2025. “Estimating Current Effective Sizes of Large Populations From a Single Sample of Genomic Marker Data: A Comparison of Estimators by Simulations.” Population Ecology 67, no. 2: 96–108.

[ece374176-bib-0134] Waples, R. S. 2006. “A Bias Correction for Estimates of Effective Population Size Based on Linkage Disequilibrium at Unlinked Gene Loci.” Conservation Genetics 7, no. 2: 167–184.

[ece374176-bib-0135] Waples, R. S. 2021. “Relative Precision of the Sibship and LD Methods for Estimating Effective Population Size With Genomics‐Scale Datasets.” Journal of Heredity 112, no. 6: 535–539. 34283240 10.1093/jhered/esab042

[ece374176-bib-0136] Waples, R. S. 2022. “What Is *Ne*, Anyway?” Journal of Heredity 113, no. 4: 371–379. 10.1093/jhered/esac023. 35532202

[ece374176-bib-0137] Waples, R. S. , and C. H. I. Do . 2010. “Linkage Disequilibrium Estimates of Contemporary ne Using Highly Variable Genetic Markers: A Largely Untapped Resource for Applied Conservation and Evolution.” Evolutionary Applications 3, no. 3: 244–262. 25567922 10.1111/j.1752-4571.2009.00104.x PMC3352464

[ece374176-bib-0138] Waples, R. S. , M. J. Ford , K. Nichols , et al. 2022. “Implications of Large‐Effect Loci for Conservation: A Review and Case Study With Pacific Salmon.” Journal of Heredity 113, no. 2: 121–144. 35575083 10.1093/jhered/esab069

[ece374176-bib-0139] Willi, Y. , T. N. Kristensen , C. M. Sgrò , A. R. Weeks , M. Ørsted , and A. A. Hoffmann . 2022. “Conservation Genetics as a Management Tool: The Five Best‐Supported Paradigms to Assist the Management of Threatened Species.” Proceedings of the National Academy of Sciences of the United States of America 119, no. 1: e2105076119. 34930821 10.1073/pnas.2105076119 PMC8740573

[ece374176-bib-0140] Williams, J. G. 2006. “Central Valley Salmon: A Perspective on Chinook and Steelhead in the Central Valley of California.” San Francisco Estuary and Watershed Science 4, no. 3: 1–398.

[ece374176-bib-0143] Yoshiyama, R. M. , F. W. Fisher , and P. B. Moyle . 1998. “Historical Abundance and Decline of Chinook Salmon in the Central Valley Region of California.” North American Journal of Fisheries Management 18, no. 3: 487–521.

[ece374176-bib-0144] Yoshiyama, R. M. , E. R. Gerstung , F. W. Fisher , and P. B. Moyle . 2001. “Historical and Present Distribution of Chinook Salmon in the Central Valley Drainage of California.” Contributions to the Biology of Central Valley Salmonids 1, no. 179: 71–176.

[ece374176-bib-0145] Zeller, D. , and D. Pauly . 2016. “Marine Fisheries Catch Reconstruction: Definitions, Sources, Methods and Challenges.” In Global Atlas of Marine Fisheries: A Critical Appraisal of Catches and Ecosystem Impacts, 12–33. Island Press.

